# Improved Visual Recognition Memory Model Based on Grid Cells for Face Recognition

**DOI:** 10.3389/fnins.2021.718541

**Published:** 2021-10-05

**Authors:** Jie Liu, Wenqiang Xu, Xiumin Li, Xiao Zheng

**Affiliations:** ^1^College of Automation, Chongqing University, Chongqing, China; ^2^School of Computer Science and Technology, Anhui University of Technology, Ma'anshan, China

**Keywords:** grid cell, visual recognition, HOG, saccade, feature

## Abstract

Traditional facial recognition methods depend on a large number of training samples due to the massive turning of synaptic weights for low-level feature extractions. In prior work, a brain-inspired model of visual recognition memory suggested that grid cells encode translation saccadic eye movement vectors between salient stimulus features. With a small training set for each recognition type, the relative positions among the selected features for each image were represented using grid and feature label cells in Hebbian learning. However, this model is suitable only for the recognition of familiar faces, objects, and scenes. The model's performance for a given face with unfamiliar facial expressions was unsatisfactory. In this study, an improved computational model via grid cells for facial recognition was proposed. Here, the initial hypothesis about stimulus identity was obtained using the histograms of oriented gradients (HOG) algorithm. The HOG descriptors effectively captured the sample edge or gradient structure features. Thus, most test samples were successfully recognized within three saccades. Moreover, the probability of a false hypothesis and the average fixations for successful recognition were reduced. Compared with other neural network models, such as convolutional neural networks and deep belief networks, the proposed method shows the best performance with only one training sample for each face. Moreover, it is robust against image occlusion and size variance or scaling. Our results may give insight for efficient recognition with small training samples based on neural networks.

## 1. Introduction

Over the last few decades, face recognition has been an active area of research in machine learning and computer vision. In the early 1990s, the historical eigenface (Turk and Pentland, 1991) was introduced. This approach recognizes faces by projecting images onto the face space and reduces dimensionality using principal component analysis (Sirovich and Kirby, 1987). Later, support vector machines (SVMs) were proposed for pattern recognition (Guo et al., 2000), and an accuracy of 97% was observed on the Cambridge ORL face database. However, SVM requires considerable time for learning big data, even for simple problems. Further, Deep Learning outperformed the SVM in accuracy in the same datasets (Gorur et al., 2019). Subsequently, non-negative matrix factorization (Guillamet and Vitria, 2002) and the AR face database were utilized to overcome the shortcomings of principal component analysis, but an accuracy of only 66.74% was observed. In 2005, the histogram of oriented gradient (HOG) approach was designed for feature extraction; this approach is currently widely used for object detection (Joshi et al., 2020). Additionally, deep belief network (DBN) (Hinton et al., 2006), which utilizes greedy layer-wise unsupervised pre-training, was used for fine-tuning the weights. This training method reduces the difficulty of learning hidden layer parameters. However, inappropriate parameter selection can lead to convergence to local optimization, which may result in extended training periods. Later in 2014, DeepFace (Taigman et al., 2014) and DeepID (Sun et al., 2014) achieved a state-of-the-art accuracy on the well-known labeled faces in the wild benchmark (Huang et al., 2008). A deep convolutional neural network (CNN) (Krizhevsky et al., 2017) can also be utilized for image classification. However, the training and execution of large-scale deep neural networks is computation-intensive even for single tasks. Large amount of training data and multiple iterations are required to ensure accuracy, which results in excessive power consumption (He et al., 2020).

With recent advances in deep learning, existing approaches of face recognition still have some limitations, such as the inability to address uncontrolled facial changes, occlusions, and scaling problems. These drawbacks may be due to the lack of a sufficient number of sample images for network training. For deep learning methods like CNN, the recognition error rate could be high for small databases (Shin et al., 2016). Recently, a computational model of visual recognition memory based on grid cells (Bicanski and Burgess, 2019) was developed, where 98 out of 99 stimuli were successfully recognized with one-shot learning, and most of the test images were recognized within four to six saccades. With real-world occlusions, an accuracy of 86.87% was obtained, which is relatively high and rarely observed. However, the model was tested on the stimuli it had learned, and the recognition performance was not satisfactory with different sets of stimuli.

In the present study, we proposed an improved computational model that utilized grid cells for face recognition. The initial hypothesis about stimulus identity was obtained by calculating the maximal similarity based on the HOG algorithm. The main contribution of this paper can be listed as follows:

1. By combining HOG algorithm with the visual recognition memory model based on grid cells, our model can provide fast global identity/hypotheses in the first saccade, which is proven to be efficient for reducing the number of saccades and resets.
2. The original computational model of visual recognition memory based on grid cells (Bicanski and Burgess, 2019) can only recognize trained/learned samples. Our model with HOG algorithm can recognize untrained test samples with much higher accuracy of face recognition.
3. Due to the existence of grid cell population, spatial relative position of feature points can be memorized through the process of eye moments during each saccade. Compared with other neural network models such as convolutional neural networks and deep belief networks, our method shows the best performance with only one training sample for each face and is robust against image occlusion, size variance, and scaling.

In subsequent sections, we present a mechanistic model based on grid cells wherein a neural circuit contributed to the visual process. Further, we introduce the HOG algorithm and our proposed method that combines the HOG algorithm with the visual recognition model. We then evaluate the performance of our model and compare it with other existing models.

## 2. Methods

### 2.1. The Original Visual Recognition Model Based on Grid Cells

**Figure 1:**
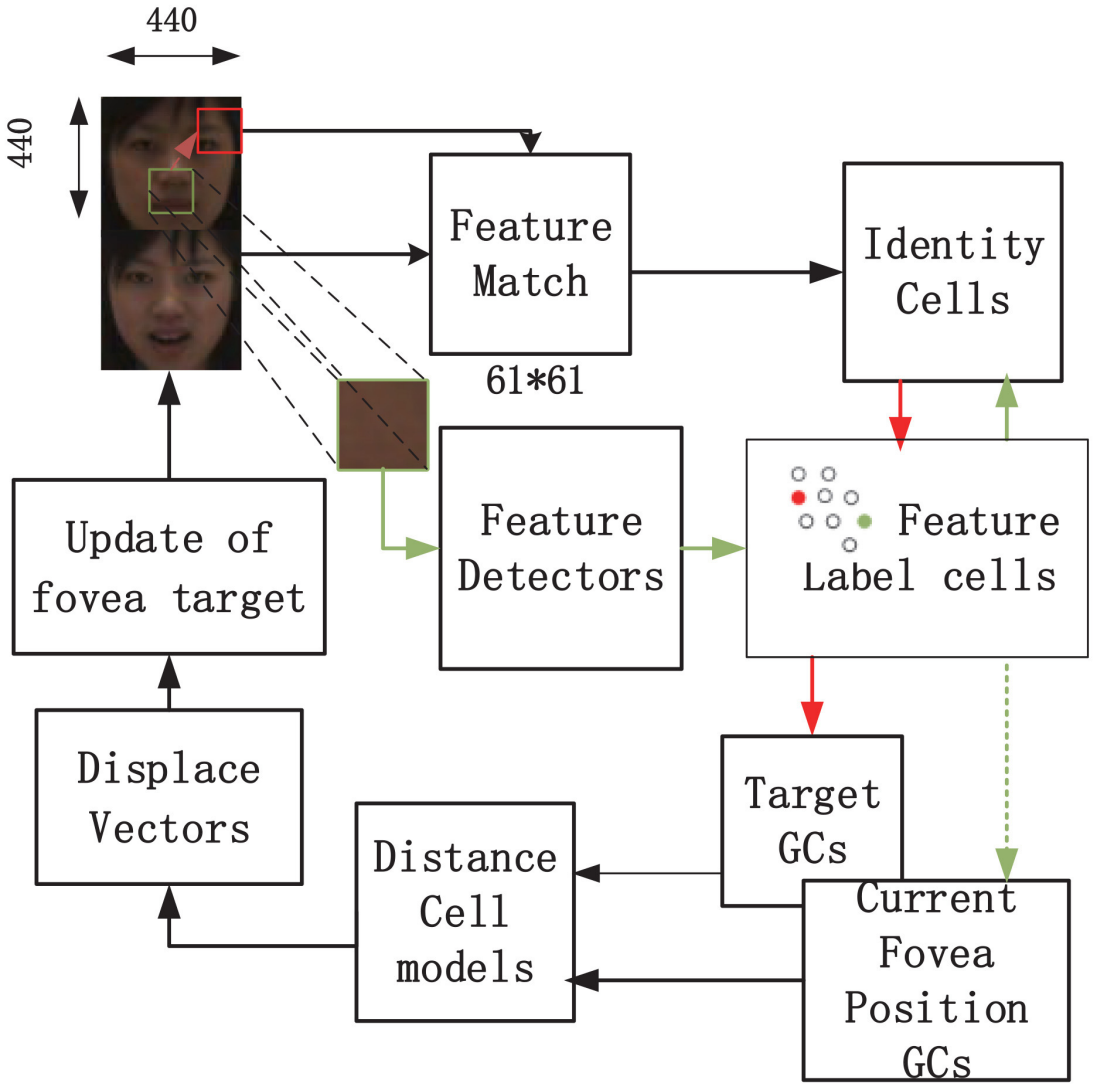
The model schematic of the visual recognition model based on grid cells, as proposed in Bicanski and Burgess (2019).

A schematic depicting the training process of the visual recognition model based on grid cells as proposed in Bicanski and Burgess (2019) is shown in Figure 1. By taking advantage of visual grid cells to encode saccade vectors, salient stimulus features were collected in advance. When learning a new stimulus, a salient position (square fovea with 61 × 61 pixels, represented as a small square in Figure 1) in the image was selected using a bottom-up attentional mechanism without prior information about the stimulus. For face recognition, the selected position had nine coordinates, including the coordinates of the corner of the eye, the tip or wing of the nose, and the corner of the mouth. The first salient features were manually selected (nine for each stimulus). Nine stimuli feature fovea matrices with a size of 61 × 61 were finally generated. Connections between feature label cells and grid cell population vectors were learned using Hebbian associations in the visual field. Thus, grid cells were used to anchor each feature of a given stimulus, and the relative positions of the anchored features encoded on all grid cells were consistent. The feature label cells corresponding to the same face identity were bi-directionally connected with the corresponding identity cell. Grid cells were implemented as standard trigger rate graphs for look-up tables. A softmax operation and a threshold in feature label cells can ensure a sparse code (Bicanski and Burgess, 2019). Some feature label cells activated the associating identity cell, generating competing hypotheses about the identity. The next saccade (i.e., the most active identity cell) is determined by the hypotheses. Furthermore, the saccade vector from the current feature fovea to the target fovea is encoded by grid cell population and calculated by the distance cell and displace cells, which are used for modeling the process of eye moments during each saccade. A similar process was repeated until the accumulated value in an identity cell reached the threshold (0.9). Finally, the leading hypothesis was accepted as the identity output. For detailed information about this model, please refer to the paper (Bicanski and Burgess, 2019).

**Figure 2:**
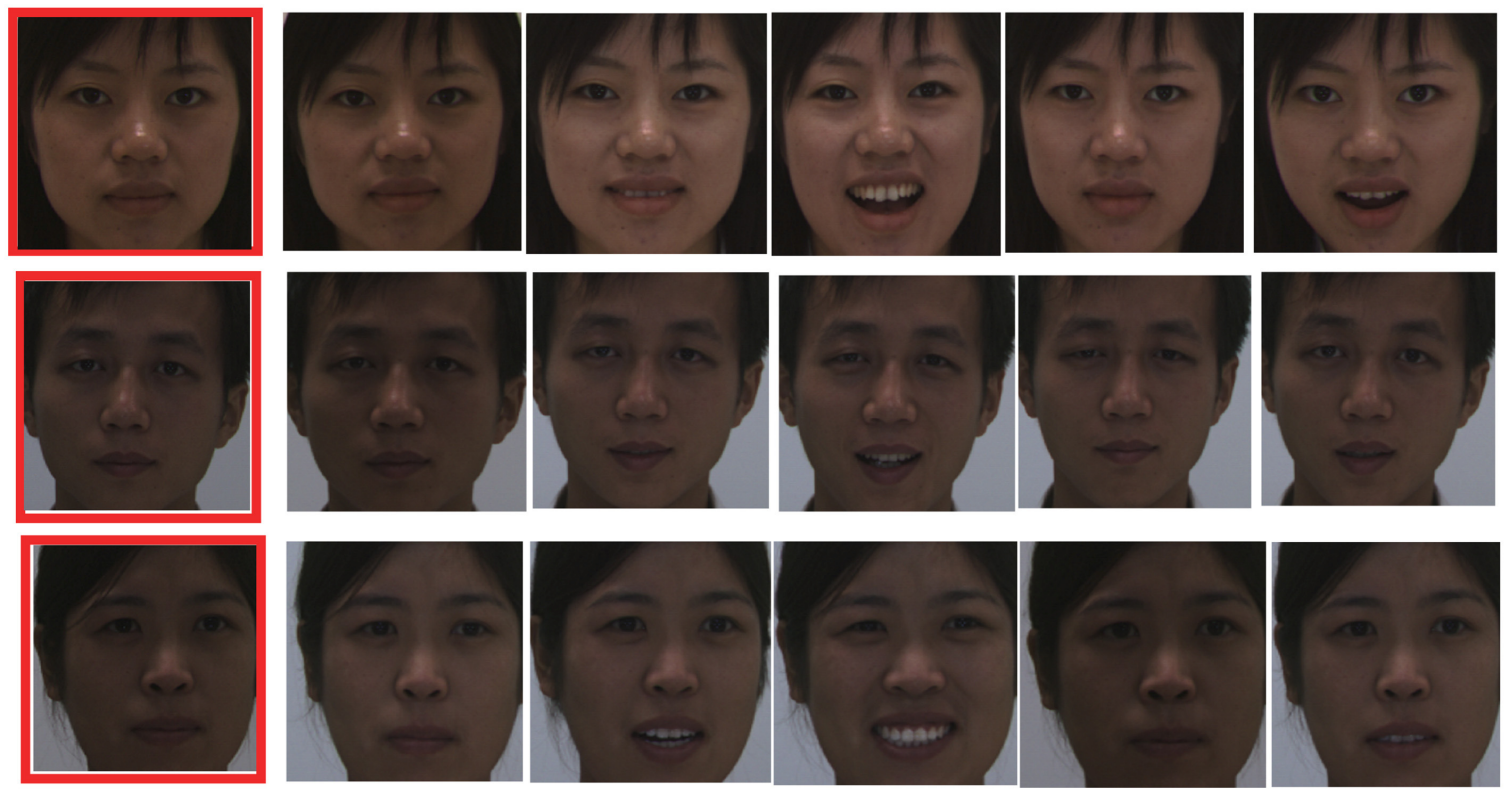
Samples of CASIA-3D Face V1 database. Only one training sample (marked in red) is used for training, the others are used for testing.

In this paper, we used the CASIA-3D face V1 database, which was collected by the Chinese Academy of Sciences Institute of Automation (CASIA). This database can be obtained from http://biometrics.idealtest.org. The face recognition data for verification were obtained from http://biometrics.idealtest.org/dbDetailForUser.do?id=8. We used the BMP1-30 dataset in CASIA-3D faces V1. From this database, we obtained 37–38 scans for each person. Additionally 2D color images and a 3D facial triangulated surface were obtained for each scan. We used images from 10 individuals with 10 front-view samples per individual. Figure 2 depicts selected samples, one image for each person (marked in red) was used for training, and the others were used for testing. The pixel size of each image was 440 x 440, which was consistent with the size map of the grid cells.

**Figure 3:**
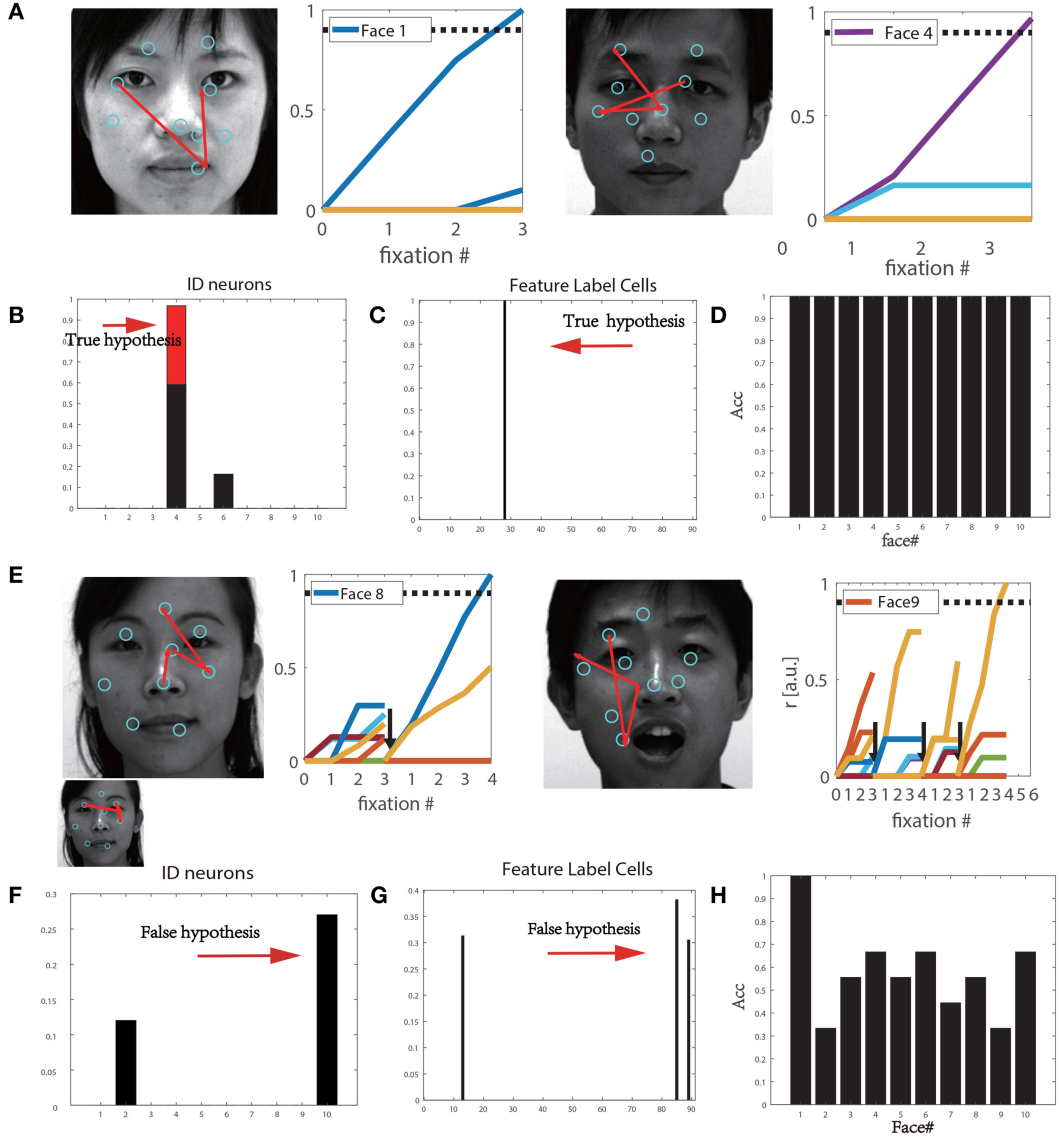
**(A)** Continuous saccades during training. **(B)** The firing rate of identity cells based on true hypothesis. **(C)** The activation rate of feature label cells based on true hypothesis. **(D)** Recognition accuracy for 10 individual types from the training set. **(E)** Continuous saccades on the test faces. **(F)** The false firing rate of the identity cells based on the false hypothesis. **(G)** The true firing rate of the feature label cells based on false hypothesis. **(H)** Recognition accuracy for 10 individual faces from the testset.

We tested the performance of the original visual recognition model proposed in Bicanski and Burgess (2019) on the CASIA-3D Face V1 database. Figure 3 depicts the performance of this model when learned (shown in Figure 3A) or unknown images (shown in Figure 3E) are used for testing. Each saccade was used to achieve a hypothesis. The saccade sequences were superimposed on the faces. The cyan circles represent the sampled features. Familiar images that were learned during training were successfully recognized within three saccades (Figures 3A,B). At the first saccade, the activated feature label cells were obtained as true predictions, as shown in Figure 3D. Figure 3C shows that the correct identity cell of the stimulus reached the given decision threshold, the stimulus was successfully recognized finally. However, when the model encountered unfamiliar images (different images of the same person), the whole performance was not satisfactory. For instance, due to an incorrect initial hypothesis, the recognition of Face#8 underwent several saccades, and was reset once before successful recognition (Figure 3E). Further, Face#9 was falsely recognized as Face#10. Therefore, an incorrect identity cell can lead to the activation of unrelated feature label cells; thus, the system initiates with a false hypothesis (Figure 3F). Here, a total of 90 new stimuli for 10 individuals were tested, and the recognition rate was only 57.8%. Actually this happened when different facial expressions were considered. Almost all feature detectors related to each stimulus were activated, which resulted in a higher number of saccades or resets and failure in feature matching. This result shows that the original model is only effective for the learned stimuli, but the recognition performance was not satisfactory for new sets of stimuli. In order to reduce the number of saccades or resets and increase recognition accuracy of unlearned testing samples, we improve this model based on the HOG algorithm as described in the following section.

### 2.2. Histogram of Oriented Gradient (HOG)

**Figure 4:**
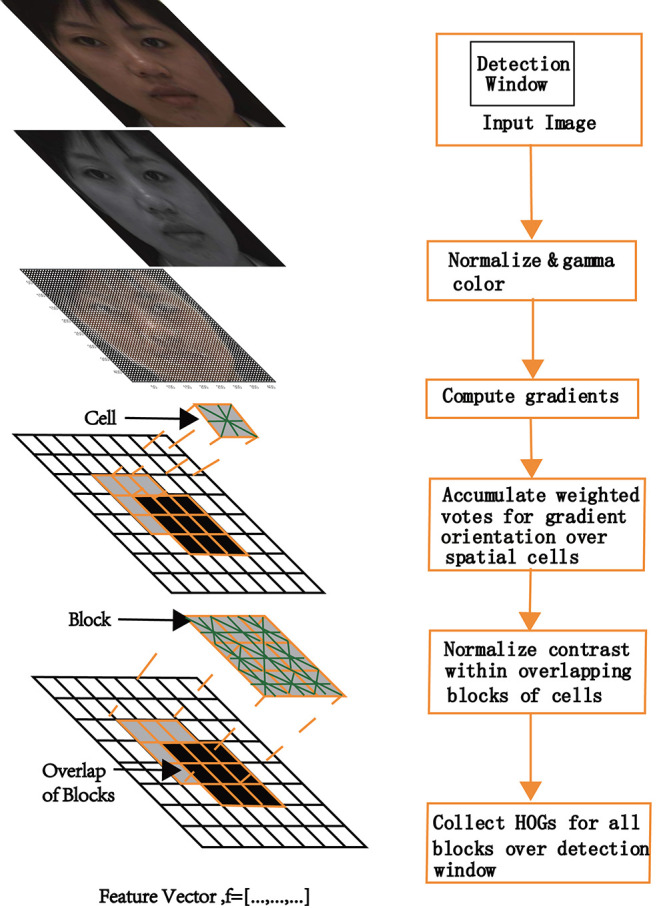
The model schematic of HOG algorithm.

Figures 4A,B shows the model schematic of HOG for face recognition. The HOG can capture the appearance and shape of objects using a distribution of local intensity gradients (Dalal and Triggs, 2005). For each input image, a horizontal and vertical gradient for each pixel was calculated using kernels (*Ix* = [−1, 0, 1], *Iy* = [−1, 0, 1]^T^). To reduce the influence of light factors, the image matrix was normalized by contrast enhancement with a gamma variation obtained from Equation (1). The horizontal and vertical gradients of the image at the pixel point G(x, y) were calculated by Equation (2). Subsequently, the image was divided into small connected areas called cells. The gradient magnitude and orientation for each cell was calculated by Equation 3 and Equation 4.


(1) $$I(x,y)=I{\left(x,y\right)}^{gamma}$$

(2) $$\{\begin{array}{l} G_{x}(x,y)=G(x+1,y)-G(x-1,y)\\ G_{y}(x,y)=G(x,y+1)-G(x,y-1) \end{array}$$

(3) $$G(x,y)=\sqrt{G_{x}{\left(x,y\right)}^{2}+G_{y}{\left(x,y\right)}^{2}}$$

(4) $$\theta (x,y)=\tan^{-1}\frac{G_{y}(x,y)}{G_{x}(x,y)}$$

Derivatives capture contours, human shadows, and texture information but weaken lighting effects. Weighted projection was performed in the gradient direction. Based on the orientation of the gradient element centered on each pixel, a weighted vote for an edge orientated histogram channel was determined, and the votes were accumulated into orientation bins over local spatial regions (cells). When detecting human beings, the best results were obtained with unsigned gradients (Dalal and Triggs, 2005), or with nine bins over 0°−180°, as depicted in this study. When the gradient orientation of the pixel was 40°−60°, the count of the third bin of the histogram increased by one. To obtain the histogram of the gradient direction of the cell, each cell pixel was weighted and projected along the gradient direction; it was subsequently mapped within the block corresponding to the calculated angle. Next, a histogram of the gradient direction of the block and window were collected. Finally, the feature vectors of all the windows were concatenated, and the HOG feature of the entire image was obtained as a feature array.

**Figure 5:**
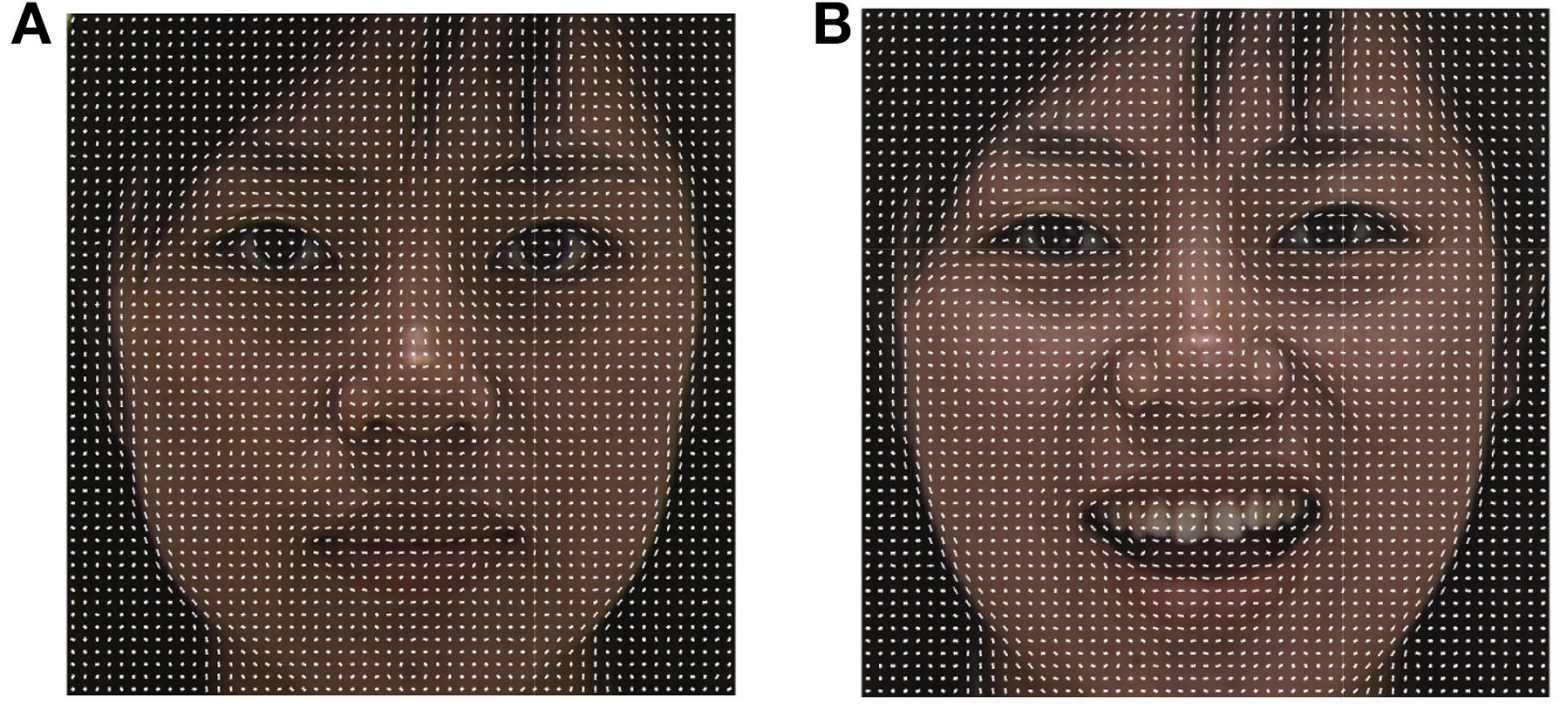
**(A)** The HOG feature vector of a training image. **(B)** The HOG feature vector of a testing image.

During the test phase, the HOG algorithm was used to extract the feature vectors of the current testing image and all of the training sets. Figures 5A,B represent the HOG feature vectors of a training image (left) and a testing image (right), respectively. Correlations between HOG features of the test face and previously trained stimuli were calculated using Equation (5). The HOG feature with the maximal correlation was considered to be belonging to the hypothetical identity cell. In this way, the first identification was determined. Since the HOG algorithm extracts the contour of objects, true hypothesis rate is high and is beneficial for quick recognition.


(5) $$corr2=\frac{\sum_{m}\sum_{n}\left(A_{mn}-\bar{A})(B_{mn}-\bar{B}\right)}{\sqrt{\left((\sum_{m}\sum_{n}{\left(A_{mn}-\bar{A}\right)}^{2}\right)\left((\sum_{m}\sum_{n}{\left(B_{mn}-\bar{B}\right)}^{2}\right)}}$$

### 2.3. Visual Recognition Model With HOG and Grid Cells (HG)

**Figure 6:**
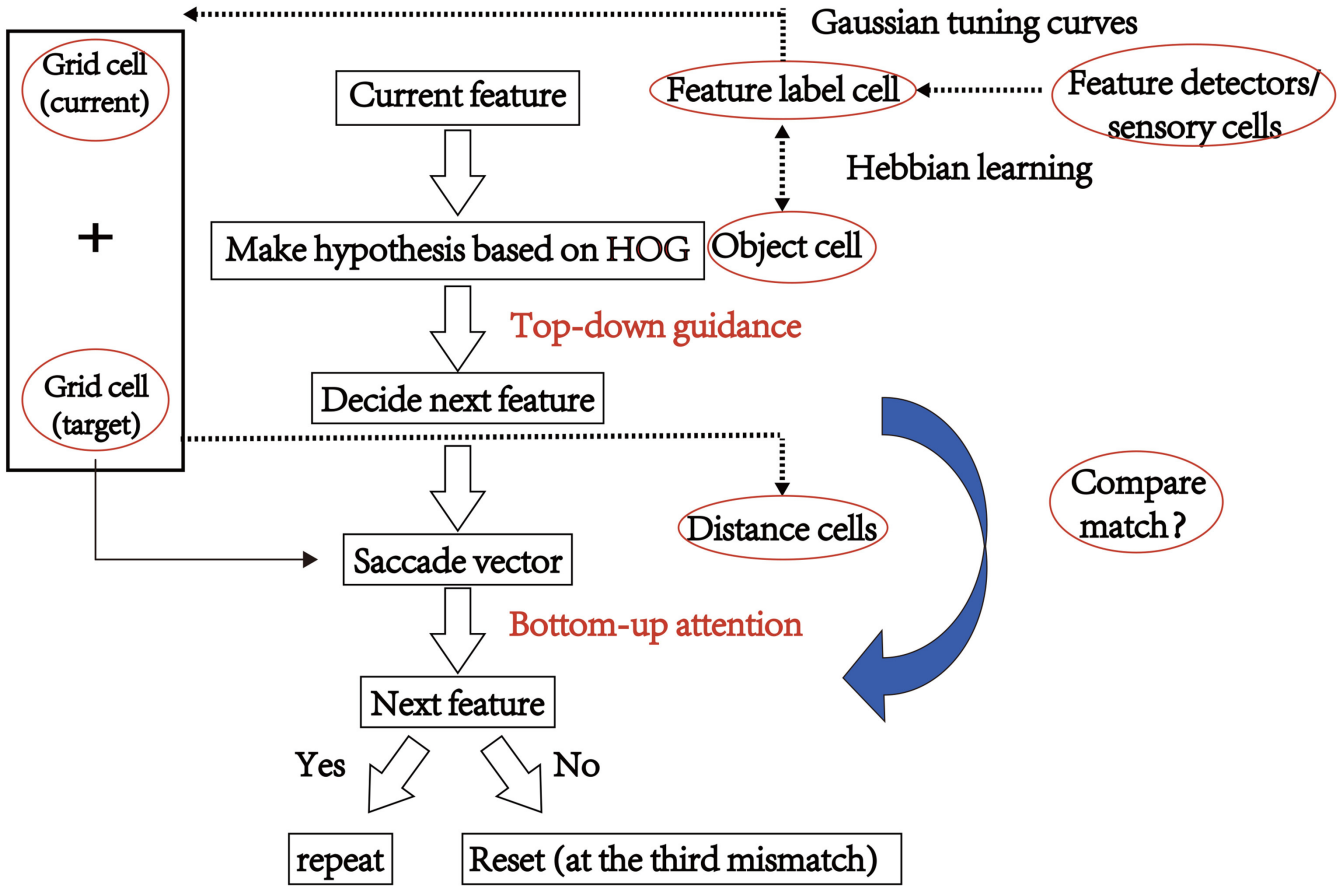
The schematic of our model based on the grid cells and HOG algorithm.

In theory, the first hypothesis should provide an identity based on the whole face instead of the local foveal, which is important for reducing reset and saccade times. Ignoring the model's biological plausibility, we improved the visual recognition model by implementing grid cells and identifying the image in the first central fovea using the HOG method to improve the accuracy of facial recognition (as shown in Figure 6). The detailed process is as follows:

Step 1: In the first action-perception cycle, correlations between the values of the HOG feature for the test face and the previously trained stimuli were calculated. The face type having the maximal correlations resulted in a hypothetical identity.
Step 2: The identity cell determined the next saccade. And the saccade vector from the current feature fovea to the target fovea was encoded by grid cell population and updated during the process of eye moments in each saccade.
Step 3: The firing rate of the hypothetical identity cell was accumulated until it reached the decision threshold. If the first hypothesis based on HOG is incorrect, the most activated feature label cells determined the associating identity cell.

Besides applying the HOG algorithm, the influence of foveal size on the recognition accuracy was also investigated. Moreover, the first salient features were manually selected (nine for each stimulus) in the original model. The entire feature extraction is time-consuming and labor-intensive for large database. Here, facial images were evenly divided into 3 × 3 blocks and the first salient features were randomly selected from these nine blocks.

Subsequently, we performed experiments to analyze the performance of our method (HG) with other algorithms. Here, two aspects were considered: normal image and scaling or occluded stimuli. In addition, to determine the validity of our model, we compared the performance of our model with other models based on both the CASIA-3D face V1 database and AR database.

## 3. Results

### 3.1. Analyses Using the CASIA-3D Face V1 Database

**Figure 7:**
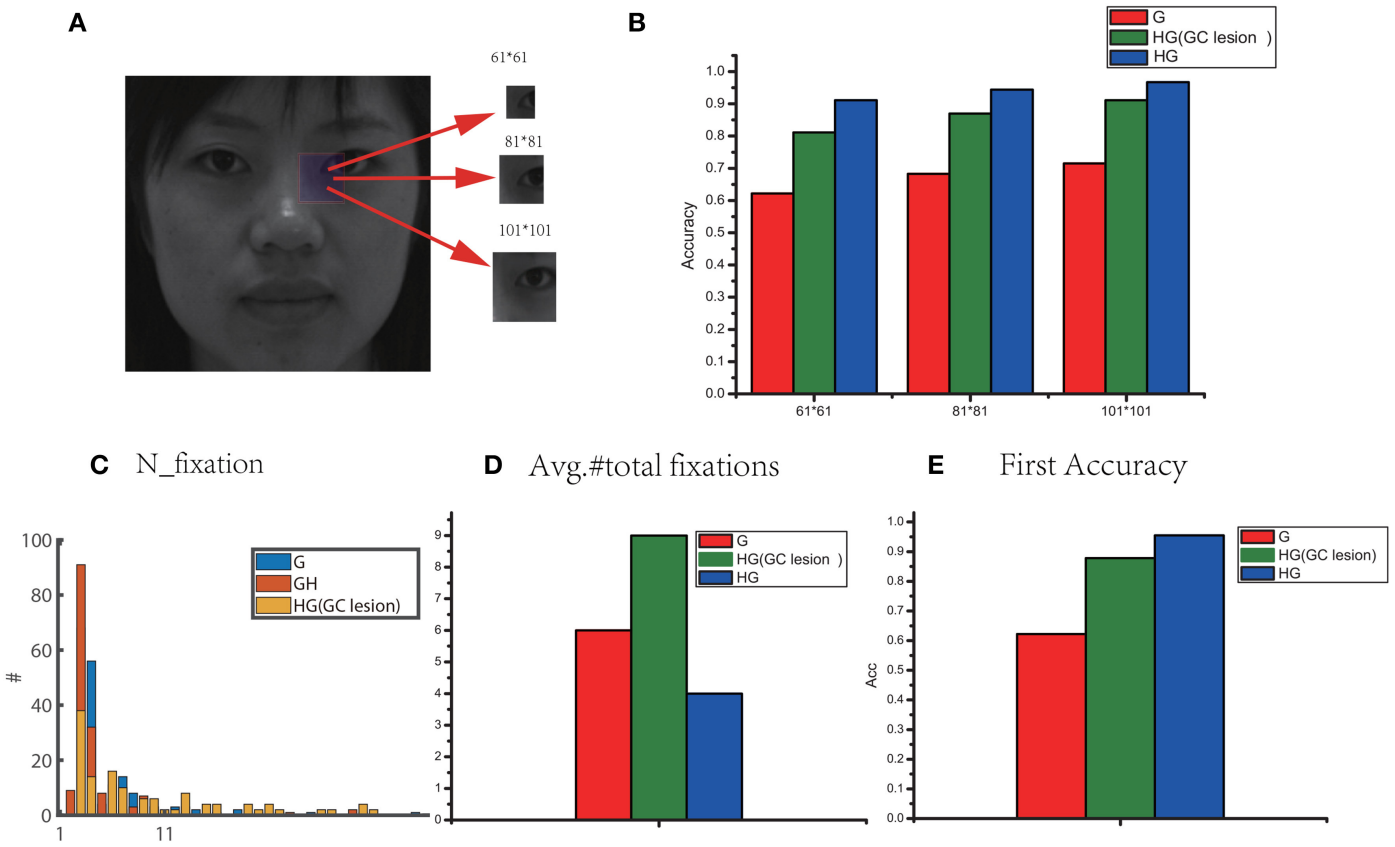
**(A)** A sample for the grayscale image (440 × 440) with different foveal sizes. **(B)** Comparisons of performance in different foveal sizes for three model: G (the original visual recognition model based on grid cells), HG (our model based on grid cells and HOG) and HG model with grid cells lesion (i.e., disconnected grid cells from the feature label cells in neurons). **(C)** The total number of fixations (including resets). **(D)** The number of saccades for recognition across all stimuli. **(E)** The first accuracy without any reset.

Figure 7A shows a representative grayscale image 440 × 440 with different foveal sizes. We compared three models: the original visual recognition model based on grid cells (G), our model (HG), and the HG model with grid cells lesion (i.e., disconnected grid cells from the feature label cells in neurons). The bar chart in Figure 7B compares the accuracy in different foveal sizes of the three models. We can see that the recognition accuracy of HG model was the highest. The performance could be improved by increasing the foveal size. Since larger foveal areas include more image pixels, this can lead to more Hebbian-learned associations between different kinds of cells (foveal array ⇒ feature label cells ⇔ identity cell) for encoding. Considering the eyes as an example, a 61 × 61 fovea framed a small portion of the eyes, but a 101 × 101 fovea sampled the entire eye area or even larger. Models with the larger fovea 101 × 101 can obtain a higher accuracy. Therefore, we used 101 × 101 fovea in our simulations. Further, Figures 7C,D show that the lesion of grid cells can greatly increase the number of fixations (including resets) and reduce the recognition accuracy. But the performance of HG with gird cell lesion is still better than the original model due to the high rate of first accuracy based on HOG (Figure 7E). HOG captures global features effectively and is helpful for hypothesis prediction by reducing the number of eye-movements and contributing to the improvement of recognition accuracy.

### 3.2. Comparisons With Different Methods

Since deep neural networks are commonly used in image recognition, we compared the accuracy of our model with a traditional CNN, a DBN with local binary patterns (LBP), namely the LD model (Murphy and Weiss, 2013), and the sparse representation-based classification (SRC) method (Wright et al., 2008).

**Figure 8:**
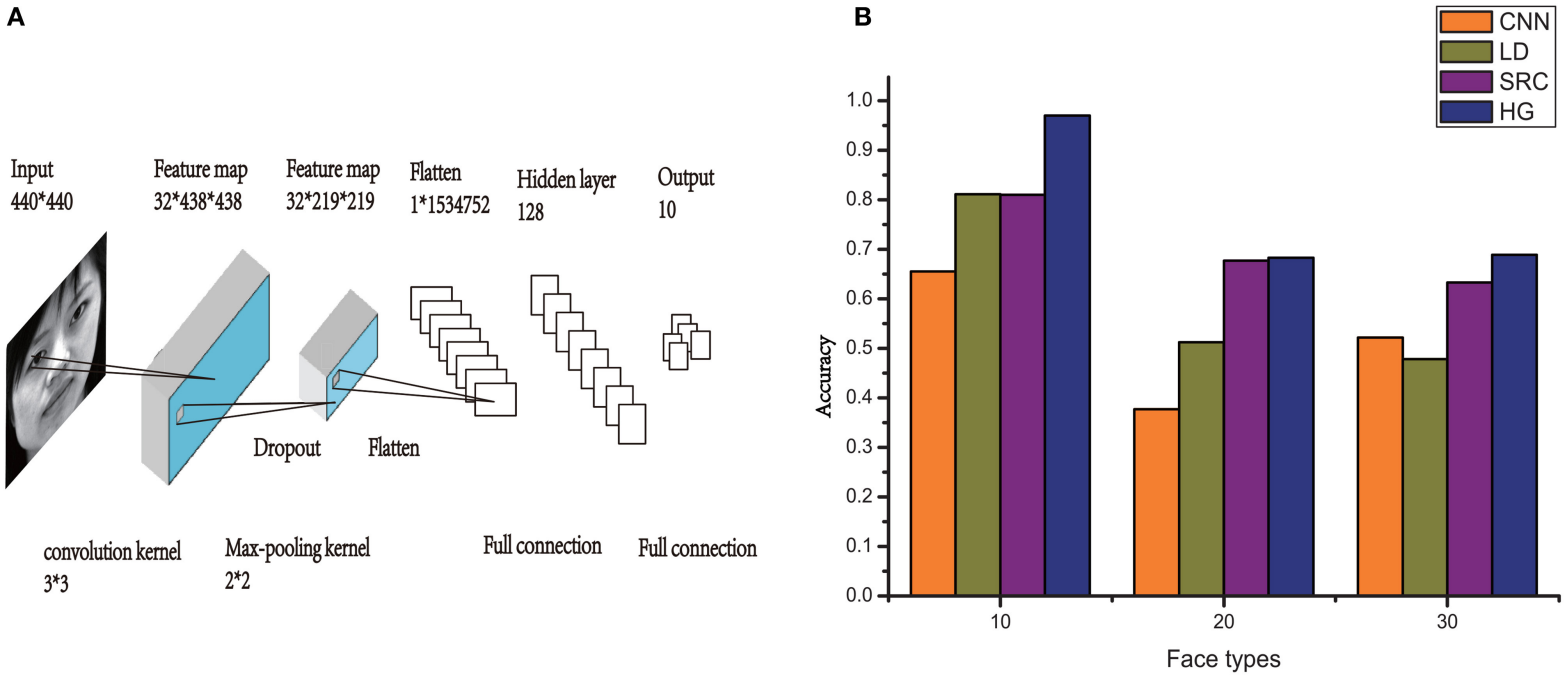
**(A)** An illustration of the architecture of CNN. **(B)** Comparison of HG (our method) with the other models on the performance of CASIA-3D face V1 database with different number of individual types.

For CNN, the Keras model (Ketkar, 2017) was used to build the topology of the CNN, which is shown in Figure 8A. Here, we created a simple 5-layer sequential connected network. The kernel size was 3 × 3, filters were set to 32, and the convolution kernel was the first layer. Each hidden neuron of the output was set to zero with a probability of 0.5.

For LBP, the image was divided into several blocks, using a 5 × 5 block, and we performed LBP for each block. Here, we chose the uniform LBP pattern. Further, texture T was defined in a local neighborhood of a monochrome texture image as the joint distribution of the gray levels of the image pixels (Ojala et al., 2002):


(6) $$T=t(g_{c},g_{0},\ldots ,g_{p-1})$$

where g denotes the gray value, *g*__*c*__ represents the center pixel in the local neighbors, and *g*_*p*_ represents the evenly spaced pixels on the radius R of the circle. Our experiment used the most commonly used parameter setting (*p* = 8, *r* = 1), and a total of 58 unique uniform types of LBP values were generated.

To verify the efficiency of the HG model, additional 20 individuals with front-view samples per individual of the CASIA-3D face V1 database are added in the experiment. The performance of our model (HG), CNN, LD, and SRC on the recognition of this database with different number of individual types (10, 20 and 30 face types) is compared in Figure 8B. We can see that although the recognition accuracy declines with the increase of individual types, HG model still outperforms the other models. When there is only one training sample, the accuracy of our model is always higher than that of the other three commonly used models.

### 3.3. Size Invariance and Occlusions

**Figure 9:**
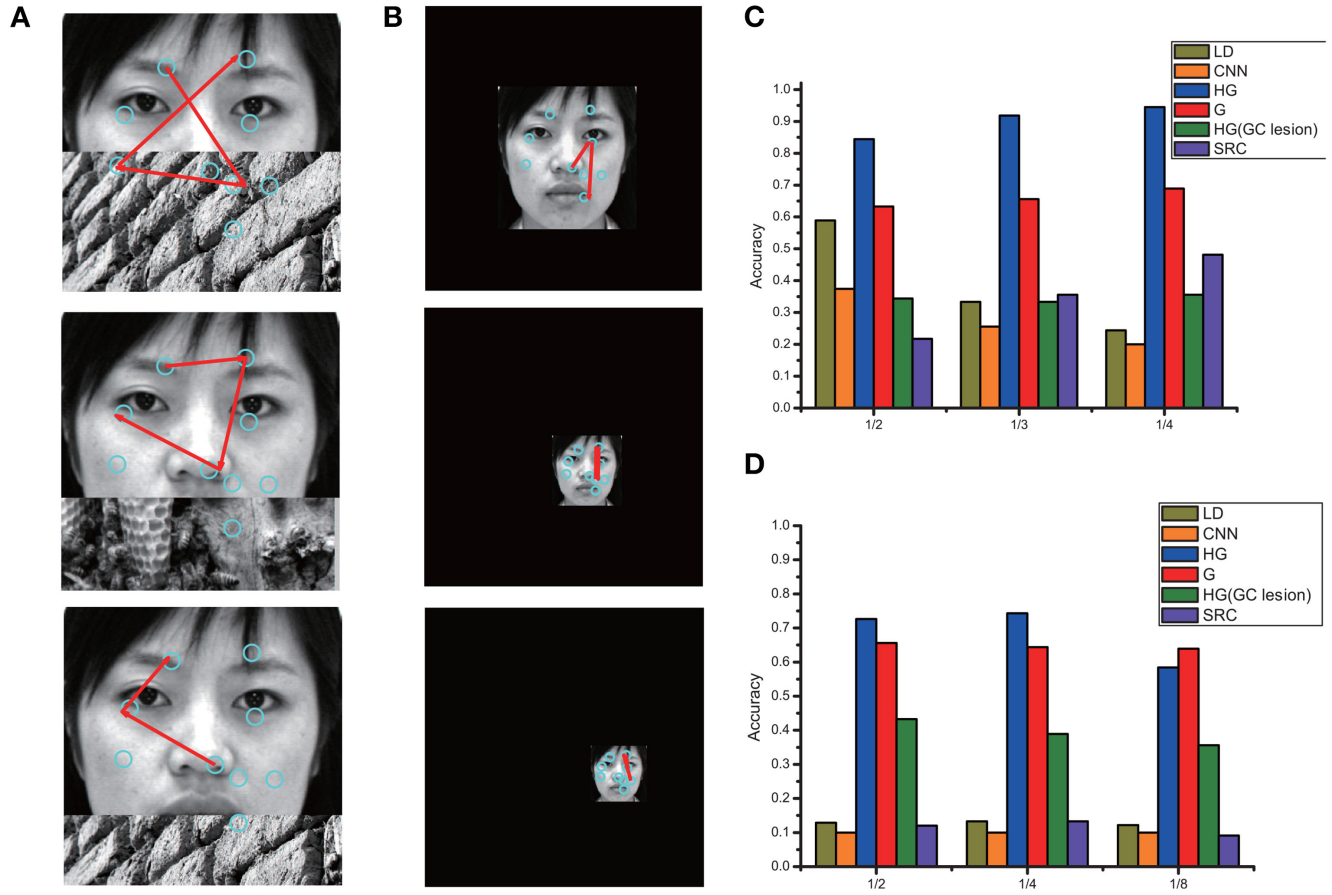
**(A)** Examples of partially occluded stimuli with different proportions of occlusion (1/2,1/3,1/4). **(B)** Examples of downscaled images with different scaling ratios (1/2,1/4,1/5). The recognition accuracy of different models on the occluded and downscaled images are compared in **(C,D)**, respectively.

Even if a stimulus is partially occluded or downscaled, the model can generate meaningful eye movement sequences (Bicanski and Burgess, 2019). We utilized two types of abnormal stimuli: real-world occlusions and downscaled images, for comparing the performance of the models. The occlusions were generated from 33 stimuli, which included objects, scenes, faces, and generic textures in equal proportions. Examples of partially occluded stimuli with different proportions of occlusion (1/2,1/3,14) are shown in Figure 9A, and downscaled images with different scaling ratios (1/2,1/4,1/5) are shown in Figure 9B. The recognition accuracies of the different models on the occluded and downscaled images have been compared in Figures 9C,D, respectively. We observed that for both cases, HG (our method) has the best performance, even when the stimuli only depicted half the face or downscaled to very small images. This result demonstrated that our model was robust to size variance and occlusions.

Considering the partially occluded stimuli, once the eye movement focuses on the occlusions, many features will mismatch. The accumulation of firing rate of the identity cell will increase slowly until the model generates the next saccade based on the previously activated one (without any hypothesis). In the case of recognition failure in a specific saccade target, most saccades will usually generate reasonable recognition because of the memory-guided eye movements based on grid cells. For a downscaled image, the visual recognition memory model can achieve size invariance by placing the downscaled image globally and correctly. The gain between the displacement vector and the output are scaled uniformly for all eye movements to sample the given features of downscaled images, leading to successful facial recognition in different size variances or encoded stimuli at different distances.

### 3.4. Analyses on the AR Face Database

**Figure 10:**
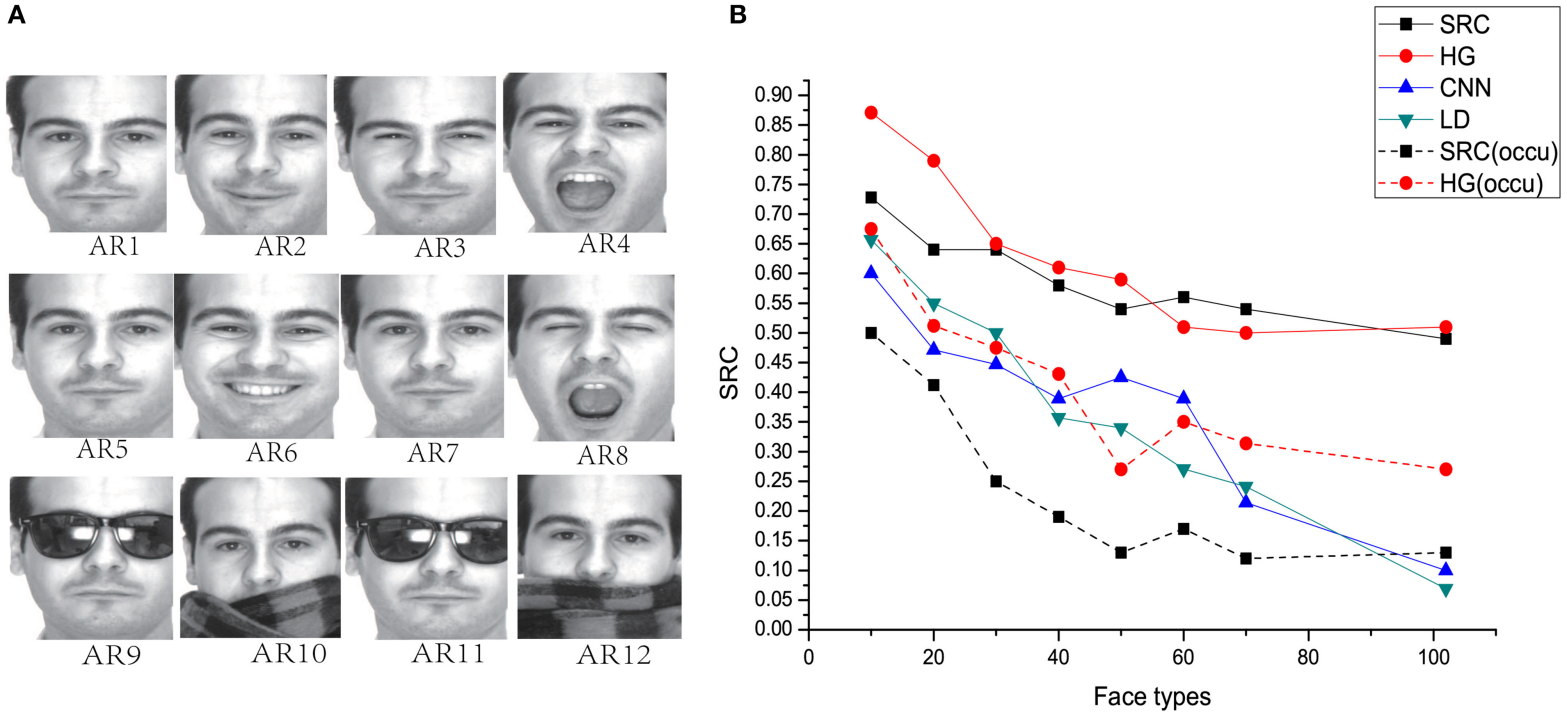
**(A)** AR face database: AR1&AR5 neutral, AR2&AR6 smile, AR3&AR7 anger, AR4&AR8 scream, AR9&AR11 sunglasses AR10&AR12 scarf. **(B)** Comparison of HG (our method) with the other models on the performance of AR face database with different number of individual types.

We verified the efficacy of our proposed model using the well-known AR database (Martinez, 1998). The AR database contains images of 116 individuals (63 males and 53 females). Some sample images are shown in Figure 10A. Sample AR1 in Figure 10A has a neutral expression, which was chosen for training. The remaining samples were reserved for testing. AR2 is a smiling face, AR3 is an angry face, and AR4 represents a screaming face. AR5-AR8 are samples with the same expression as that of AR1–AR4. The original image had a size of 768 × 576 pixels with 24-bit color resolution. AR9–AR12 are samples with sunglasses and scarves. To avoid the influence of the external background, we only considered face regions by training separate detectors for the original images (Hu and Ramanan, 2017). The pictures were cropped and reshaped to 440 × 440 pixels.

Using seven training samples (AR2-AR8), the SRC method achieved a recognition rate of 92% with 540 dimensional features for all individuals. Here, we used only one sample (AR1) for training, and AR2-AR8 were used for testing. Comparisons among these models are shown in Figure 10B with different numbers of individuals. We observed that HG model still shows the best performance on this database. Although the outperformance of our model compared with SRC is not obvious when the number of face types is larger than 60, our model is much more robust than SRC when images are occluded (as shown in Figure 10B, dash lines).

## 4. Conclusions

In this study, an improved computational model via grid cells for face recognition was proposed. Here, the initial hypothesis about stimulus identity was obtained by calculating the maximal similarity based on the HOG algorithm. The HOG descriptors can effectively capture the features of the sample edge or gradient structure. Utilizing this model, most of the test samples were successfully recognized within three saccades. The false hypothesis and average fixations for successful recognition were reduced. With only one training sample for each face, our method outperformed the original model, SRC model and deep learning neural networks like CNN and DBN. Since there is only one training sample for each face type in the experiments of this paper, synaptic weights of CNN and DBN can not be fully updated, leading to insufficient feature detections. Adding more hidden layers will not obviously improve the performance but greatly increase time consumption. Actually, deep learning method strongly depend on big training data. In the case of single training sample for each image, these deep learning models do not perform well and can be easily disturbed by image occlusion, size variance, and scaling (as shown in Figures 8–10). Although incorporating HOG into this visual recognition memory model voids its biological plausibility, the main purpose of this study is to improve the accuracy of facial recognition based on grid cells, instead of maintaining the biological mechanisms involved in facial recognition. Our results may give insight for efficient recognition with small training samples based on brain-inspired neural networks.

## Data Availability Statement

The original contributions presented in the study are included in the article/supplementary material, further inquiries can be directed to the corresponding author/s.

## Author Contributions

JL prepared the data and wrote the manuscript. WX helped for data collecting. XL gave the idea and planed the whole work. XZ provided many meaningful suggestions. All authors contributed to the article and approved the submitted version.

## Funding

This work is supported by the Natural Science Foundation of Chongqing (No. cstc2019jcyj-msxmX0154) and Program for Synergy Innovation in the Anhui Higher Education Institutions of China (No. GXXT-2019-025).

## Conflict of Interest

The authors declare that the research was conducted in the absence of any commercial or financial relationships that could be construed as a potential conflict of interest.

## Publisher's Note

All claims expressed in this article are solely those of the authors and do not necessarily represent those of their affiliated organizations, or those of the publisher, the editors and the reviewers. Any product that may be evaluated in this article, or claim that may be made by its manufacturer, is not guaranteed or endorsed by the publisher.
